# The *Glaciozyma antarctica* genome reveals an array of systems that provide sustained responses towards temperature variations in a persistently cold habitat

**DOI:** 10.1371/journal.pone.0189947

**Published:** 2018-01-31

**Authors:** Mohd Firdaus-Raih, Noor Haza Fazlin Hashim, Izwan Bharudin, Mohd Faizal Abu Bakar, Kie Kyon Huang, Halimah Alias, Bernard K. B. Lee, Mohd Noor Mat Isa, Shuhaila Mat-Sharani, Suhaila Sulaiman, Lih Jinq Tay, Radziah Zolkefli, Yusuf Muhammad Noor, Douglas Sie Nguong Law, Siti Hamidah Abdul Rahman, Rosli Md-Illias, Farah Diba Abu Bakar, Nazalan Najimudin, Abdul Munir Abdul Murad, Nor Muhammad Mahadi

**Affiliations:** 1 School of Biosciences and Biotechnology, Faculty of Science and Technology, Universiti Kebangsaan Malaysia, Bangi, Selangor, Malaysia; 2 Institute of Systems Biology, Universiti Kebangsaan Malaysia, Bangi, Selangor, Malaysia; 3 Malaysia Genome Institute, Jalan Bangi Lama, Kajang, Selangor, Malaysia; 4 Department of Biosciences Engineering, Faculty of Chemical & Natural Resources Engineering, Universiti Teknologi Malaysia, Skudai, Johor, Malaysia; 5 School of Biological Sciences, Universiti Sains Malaysia, Penang, Malaysia; University of Cambridge, UNITED KINGDOM

## Abstract

Extremely low temperatures present various challenges to life that include ice formation and effects on metabolic capacity. Psyhcrophilic microorganisms typically have an array of mechanisms to enable survival in cold temperatures. In this study, we sequenced and analysed the genome of a psychrophilic yeast isolated in the Antarctic region, *Glaciozyma antarctica*. The genome annotation identified 7857 protein coding sequences. From the genome sequence analysis we were able to identify genes that encoded for proteins known to be associated with cold survival, in addition to annotating genes that are unique to *G*. *antarctica*. For genes that are known to be involved in cold adaptation such as anti-freeze proteins (AFPs), our gene expression analysis revealed that they were differentially transcribed over time and in response to different temperatures. This indicated the presence of an array of adaptation systems that can respond to a changing but persistent cold environment. We were also able to validate the activity of all the AFPs annotated where the recombinant AFPs demonstrated anti-freeze capacity. This work is an important foundation for further collective exploration into psychrophilic microbiology where among other potential, the genes unique to this species may represent a pool of novel mechanisms for cold survival.

## Background

The extreme cold environments of the polar, deep sea and alpine regions are inhabited by psychrophilic microbial life that have evolved physiological strategies for cold adaptation that include the production of antifreeze proteins, modulation of enzyme kinetics and preservation of membrane fluidity [1, 2]. Cowan and colleagues [3] have reviewed the impact of ‘omic’ technologies towards understanding the molecular mechanisms of psychrophilic adaptation in which they also presented a list of completed and unfinished psychrophilic genomes. The early genomes of the psychrophilic microbes [4–7] were able to provide a glimpse into content that were unique for cold temperature survival and these were thus able to provide an understanding into role of genome evolution in cold adaptation [8].

In addition to genome and omics acquired insights, biochemical investigations into enzyme function at low temperatures also contribute to a collective understanding how molecular adaptations [9, 10] can be translated to a physiological psyhrophilic lifestyle. For psychrophilic microorganisms, the absence of a physical insulative barrier to keep out the cold meant that adaptation had to be physiological and implemented at the cellular and molecular level. These adaptations include the production of antifreeze proteins or cryoprotectants, changing the fluidity of its membrane and the modification of enzyme properties [2].

Cellular freezing that could damage the capacity for intracellular activity and change the osmotic level need to be avoided, and this could be achieved by the production of exopolysaccharides (EPS) that function as cryoprotectants by trapping water, nutrients and metal ions thus facilitating biofilm formation around the cell [11]. Furthermore, EPS can also lower the freezing point and ice nucleation temperature of the water [2]. In addition, the presence of free radicals outside the cells could be protected by the formation of trehalose disaccharide which can also stabilize the cellular membrane under extreme cold condition [12].

The rigidity of the membrane at freezing temperatures can be overcome by increasing the polyunsaturated fatty acid (PUFA) content [1]. This would hypothetically make genes encoding fatty acid desaturases (FAD) that are involved in the addition of PUFA an important characteristic of cold adaptation and many studies have indeed shown increased expression of genes encoding FAD at lower temperatures [13, 14]. Comparative studies also show that general membrane transport proteins such as peptide transporters, which are involved in the uptake of nutrients and membrane peptides, are also up-regulated at lower temperatures [15].

Furthermore, molecular studies shown that psychrophilic enzymes increased their structural flexibility in freezing temperatures due to the increased content of specific amino acids such as glycine [16] and this is notably so near the enzyme catalytic sites in order to increase its local mobility [17]. In addition, the lysine-to-arginine ratios also increased thus lowering hydrogen bonding and salt bridge formation [18, 19].

To date, most cold-adapted enzymes have been discovered and sourced from bacteria [9, 20, 21] and archaea [22, 23]; even so, antifreeze proteins and several cold adapted metabolic enzymes have been reported for several fish species [24, 25]. Most ice binding proteins are known to be monomeric, however, Lee et al 2012 reported a novel dimeric example from the Arctic yeast *Glaciozyma* sp. (formerly known as *Leucosporidium* sp.) that had a complex ice binding site which may allow interactions with multiple faces of the ice crystal [26]. Another report for *Glaciozyma* sp. ice binding protein presented its capacity to provide antioxidative effects [27].

Analyzing the genome of a psychrophilic eukaryotic microbe will provide insights into the cold adaptation strategies that are independent of the significant insulative barrier mechanisms available to vertebrate eukaryotes. In this work, we report the first genome sequence for a pyschrophilic yeast, *Glaciozyma antarctica*. Several physiological pyschrophily mechanisms for microbes that include the production of antifreeze proteins (AFP), an increased proportion of unsaturated fatty acids in the membrane for maintenance of membrane fluidity, cold adapted enzymes and the presence of cryoprotectant sugars and polyols have been reviewed by D’amico et al. [1]. In this paper, we show the presence, in *G*. *antarctica*, of such previously reported cold adaptation mechanisms by first identifying components of the genome that encode antifreeze proteins (AFPs) and fatty acid desaturases (FADs). Having identified the relevant genes, we were then able to uncover temperature-related variations of gene expression levels for the different AFPs and FADs encoded in the *G*. *antarctica* genome by quantitative real-time polymerase chain reaction (RT-qPCR) experiments.

It is most probable that a combination of mechanisms are employed by *G*. *antarctica* for cold temperature survival and growth as has been previously reported by Blanc et al [28] for the psychrophilic microalga *C*. *subellipsoidea*. Comparative analyses were further able to contrast features of the genome that were possibly due to *G*. *antarctica’s* psychrophilic lifestyle that were absent in non-psychrophilic yeasts but present in other, more distantly related, psychrophiles. Our genome characterization and subsequent validation experiments provide insights into an extensive array of defenses deployed to protect the cell against extreme persistently cold environments.

## Results and discussion

### Genome content and structure

The *Glaciozyma antarctica* PI12 draft genome sequence (GenBank accession ASRT0000000) was generated using a combination of 454 pyrosequencing (2.33 Gb; 117X coverage) and Sanger sequencing of 12,948 fosmid sequences (8.9 Mb). The 20,033,549 bps were assembled into 21 gap-free scaffolds ranging from 2.7 kb to 2.28 Mb. Gene predictions identified 7857 putative protein coding genes (CDS) that make up 59.2% of the genome sequence, 79 tRNA genes and 3 rRNA genes. The ~20 Mb *G*. *antarctica* genome is similar in size to those of two other basidiomycetes with published genome sequences *Cryptococcus neoformans* [29] and *Ustilago maydis* [30] (Table 1) although it has a considerably higher G+C content of 60% (Table 1).

**Table 1:** Summary of the *G*. *antarctica* genome in comparison to other fungi and bacterial representative genomes.

Species	Features	Size (Mb)	G+C content (%)	Scaffolds(sc)/ Chromosome(chr)	Genes	tRNAs	rRNAs
**Fungi**	***G*. *antarctica***	20	60	21 sc	7,857	79	3
	***P*. *destructans***	30.68	49.98	1847 sc	9,153	124	26
	***C*. *neoformans***	19	48.6	14 chr	6,572	141	NA
	***S*. *cerevisiae***	19	38.3	16 chr	6,575	275	25
	***C*. *thermophilum***	28.3	52.6	21 sc	7,165	243	NA
	***M*. *thermophila***	38.74	51.1	7 sc	9,110	436	51
**Bacteria**	***C*. *psychrerythraea***	2.84	38	1 chr	4,393	88	28
	***S*. *baltica***	5.29	46.3	1 chr	4,412	104	31
	***E*. *coli***	5.5	50.5	1 chr	5,204	105	22
	***S*. *aureus***	2.76	32.9	1 chr	2,460	59	19
	***C*. *kronotskyensis***	2.84	35.1	1 chr	2,470	47	9
	***A*. *mobile***	2.16	48	1 chr	2,008	49	6

A separate set of EST sequences was also used for assembly validation and further functional annotation of the predicted gene models. The EST sequences were generated from nine cDNA libraries that were constructed from mRNA extracted from variations of temperature (-12°C, 0°C, 15°C), media (YPD and YNB see Table A in S1 File) and growth phases (lag, exponential, post-diauxic, stationary phases). The combined EST contigs yielded 7,369 unique transcripts consisting of 4,203 consensus and 3,166 singleton sequences. The majority (67%) of the predicted coding regions from the genome assembly were matched to the ESTs leaving 726 predicted genes with known homologs without assigned ESTs. The remaining 2920 (37%) of the predicted genes were classified as hypothetical proteins.

In order to acquire further insights into cold adaptation mechanisms that could be extracted from the genome sequence data, the genome was analyzed with the specific intent of (i) identifying unique components of the *G*. *antarctica* genome that are not present in the available genome sequences of other yeasts; (ii) identifying components of the genome that were shared with other psychrophiles but are absent in other yeasts; and (iii) identifying orthologs of proteins that have been reported to be involved in cold adaptation. We were then able to select targets from these analyses for validation by either mapping their results back to ESTs that were products of cDNA libraries generated from three different *G*. *antarctica* culture temperatures (15°C, 0°C, -12°C), or via RT-qPCR analysis of the mRNA extracted from *G*. *antarctica* at optimal culture temperatures (15°C) and those that simulated cold stress (0°C and -12°C).

### Intergenic, intronic and snoRNA sequences

**Fig 1:**
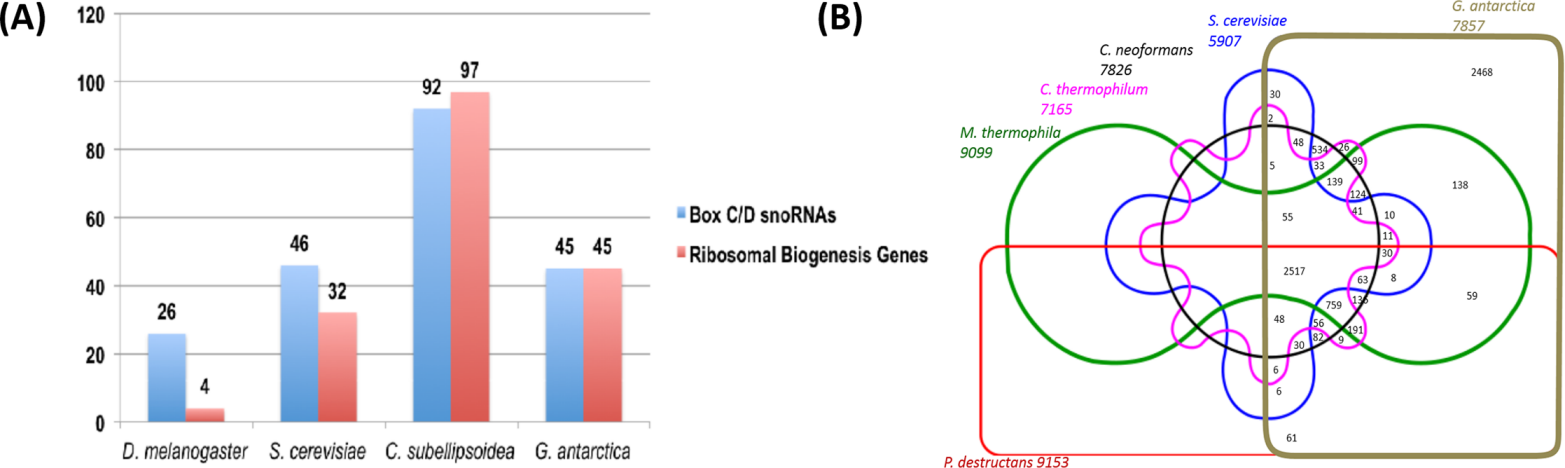
snoRNA sequence and yeast conserved orthologs. (A) comparison of predicted box C/D small nucleolar RNA (snoRNA) and ribosomal biogenesis genes in *Drosophila melanogaster*, *Saccaromyces cerevisiae*, *Coccomyxa subellipsoidea* and *Glaciozyma antarctica*, (B) comparison of predicted conserved orthologs between *G*. *antarctica*, *Pseudogymnoascus destructans*, *Cryptococcus neoformans*, *Saccaromyces cerevisiae*, *Chaetomium thermophilum* and *Myceliophthora thermophila*.

Intergenic and intronic sequences represent 70.85% of the entire *G*. *antarctica* genome. Similar intronic and intergenic contents have been observed in related yeast counterparts, with 63.85% in *Saccharomyces cerevisiae* (S288c Chromosome I-XVI), 71.66% in *Schizosaccharomyces pombe* (972h- Chromosome I-III) and 73.65% in *Cryptococcus neoformans* (var. neoformans JEC21 Chromosome 1–14). A total of 45 box C/D small nucleolar RNA (snoRNA) genes were predicted by analysing the extracted intronic and intergenic sequences (Fig 1A). SnoRNAs are small RNA molecules that guide the chemical modifications of other RNAs by enzymes such as methyltransferases (for box C/D snoRNAs) and pseudouridylases (for box H/ACA snoRNAs [31, 32]. By using the predicted 18S and 25S rRNA sequences as possible modification targets, we identified nine potential methylation sites and two potential pseudouridylation sites for snoRNA marking.

Further analysis revealed that each of the 45 putative box C/D snoRNA genes form pairings with ribosomal RNA processing genes in one of their flanking regions. We compared this finding to box C/D snoRNA genes that we annotated for the polar microalga *C*. *subellipsoidea* from the genome sequence reported by Blanc et al. [33] and discovered that a similar ratio and arrangement is present in the *C*. *subellipsoidea* genome. Out of the 97 ribosomal biogenesis genes found, 92 were predicted to have box C/D snoRNAs residing either on their upstream or downstream flanks (Fig 1A). This is equivalent to about 94.8% of the total ribosomal biogenesis genes in *C*. *subellipsoidea*. In contrast, only 32 out of 46 box C/D snoRNA genes with similar ribosomal RNA processing gene pairings have been reported in *Saccharomyces cerevisiae* [34–36], while only 4 out of 26 box C/D snoRNA genes are so arranged in *Drosophila melanogaster* [37, 38] (Fig 1A). This increased number of ribosomal protein biogenesis genes in ratio to the number of C/D snoRNA genes perhaps reflect the existence of a mechanism to compensate for possibly slower rates of protein synthesis in a psychrophilic environment.

### Yeast conserved orthologs and psychrophile-specific genes

The identification of orthologs shared between *G*. *antarctica* and five other available fungal genomes that are mesophilic yeast (*Saccharomyces cerevisiae* and *Cryptococcus neoformans*), thermophilic fungi (*Myceliopthora thermophile* and *Chaetomium thermophilum*) and a psychrophilic fungi (*Pseudogymnoascus destructans*) enabled us to contrast the contents for these genomes thus identifying *G*. *antarctica* genes absent in the more closely related yeasts but present in *P*. *destructans* and therefore possibly associated to psychrophily. These comparisons revealed that out of the predicted 7857 putative protein coding sequences (CDS) for *G*. *antarctica*, 31.4% of the genes were unique to the *G*. *antarctica* genome for this particular comparison set while the remaining 68.6% had detectable orthologs in the other five fungal genomes (Fig 1B).

Several protein families and domains have been associated to cold stress responses. These proteins are known to function either as transcription factors (cold shock domains), and/or chaperones (cold shock domains, heat shock proteins and peptidyl prolyl isomerases). Among the genes absent in the non-psychrophilic yeast genomes are four that encode for proteins containing cold-shock domains (GAN_01_006, GAN_16_627, GAN_16_676, GAN_16_853). Proteins of this class are known to bind nucleic acids as transcription factors or RNA chaperones [39, 40] and typically contain a conserved cold shock domain (CSD) carrying RNA recognition motifs. The arrangements of the CSD were different for each of these four potential cold-shock response proteins (CSP), suggesting that they are parts of proteins that perform different roles and possible products of convergent instead of divergent evolution. An ortholog for GAN_16_627 was not found in the *C*. *subellipsoidea* genome while the other three CSPs had detectable orthologs. Three of the CSPs had one cold shock domain with each containing the RNA recognition motifs 1 and 2. Compared to the other three CSPs, GAN_16_627 was found to contain two adjacent cold shock domains with one domain being free of the RNA recognition motifs.

A total of nineteen peptidylprolyl isomerases (PPIase) were also annotated. These enzymes interconvert the cis and trans isomers of proline peptide bonds and can thus effect an increase in the rate of protein folding. PPIases that are active at low temperatures are therefore important to maintain protein folding rates. Of the nineteen PPIase encoding ORFs, one was not found in any of the other four genomes compared (GAN_04_354). Six heat shock protein genes (GAN_02_075, GAN_02_254, GAN_08_430, GAN_13_463, GAN_16_081 and GAN_17_157) also did not have homologs in the three other yeast genomes compared. These genome level comparisons further identified 299 orthologs in all three non-psychrophilic yeast genomes that are missing in *G*. *antarctica*.

### Anti-freeze proteins are expressed in response to cold temperatures

AFPs are known to inhibit ice growth and recrystallization thus enabling organisms producing these proteins to survive at temperatures below 0°C. The affinity of AFPs towards ice can depress the freezing point of a solution in a non-colligative process known as thermal hysteresis (TH) as well as inhibiting ice recrystallization (RI) and thus preventing the formation of larger ice grains. *G*. *antarctica* was discovered to secrete at least one antifreeze protein into the culture medium when grown at low temperatures (< 10°C) [41]. The search for homologs to known AFPs revealed a total of nine non-paralogous AFP genes designated as *GaAFP1-GaAFP9*. The nomenclature for the AFPs we describe here is based on the molecular weight, with *GaAFP1* being the smallest, and *GaAFP9* the heaviest. The AFPs of *G*. *antarctica* also did not have any sequence homologs to any proteins encoded in the *C*. *subellipsoidea* genome published by Blanc et al [28]. GaAFP1 (GAN_05_237) was identified as the AFP previously reported by Hashim et al [41]. A wider BLAST search against the non-redundant database at GenBank revealed that homologs of these AFPs have been documented in other psychrophiles. It was also discovered that scaffolds 5 and 16 each contained three adjacently located AFP genes; GAN_05_236 (*GaAFP8*), GAN_05_237 (*GaAFP1*), and GAN_05_238 (*GaAFP7*) in scaffold 5 while GAN_16_650 (*GaAFP3*), GAN_16_651 (*GaAFP4*) and GAN_16_667 (*GaAFP5*) were in scaffold 16.

**Fig 2:**
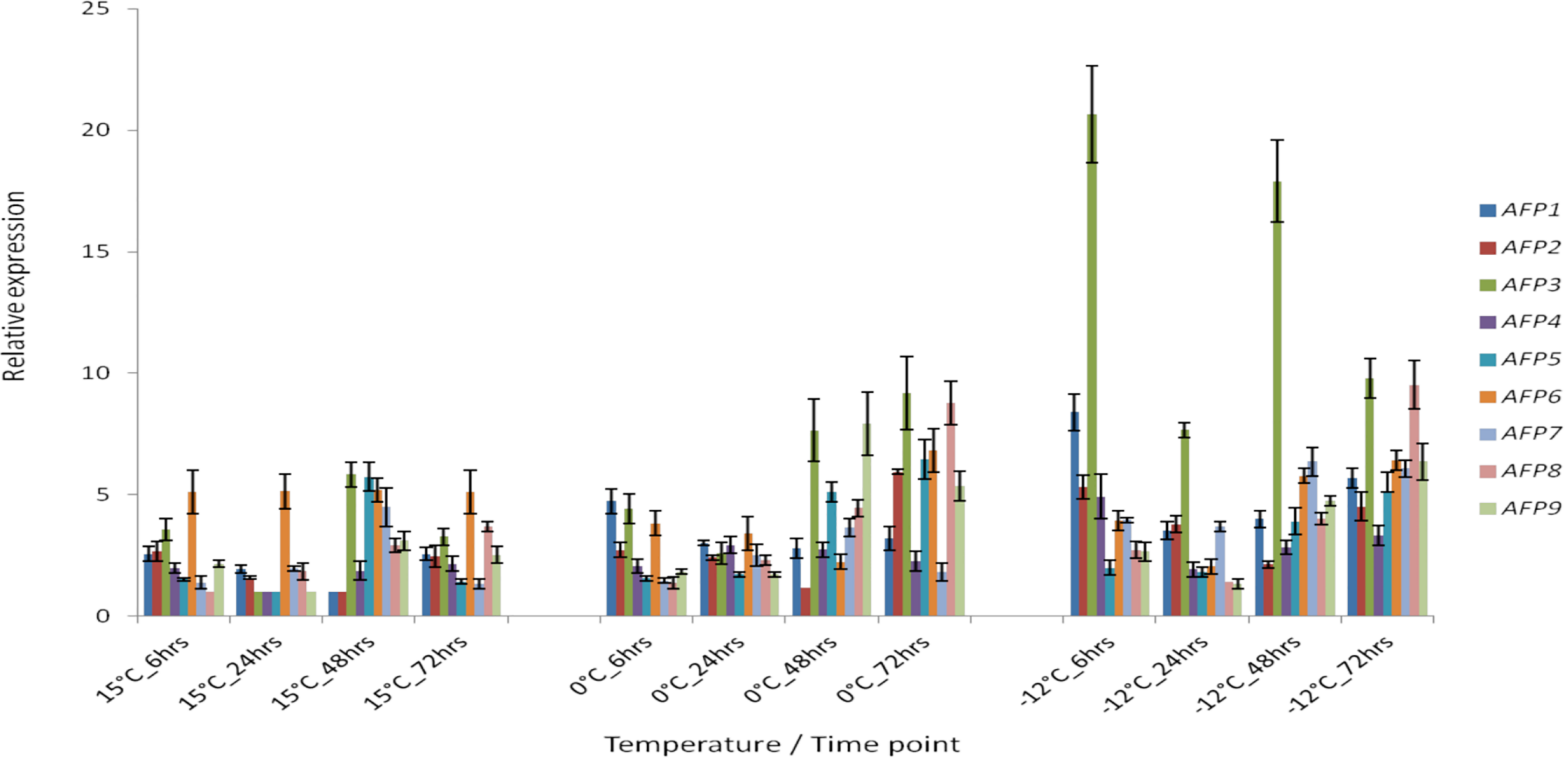
Relative expression profiles of nine GaAFP genes at different temperatures; 15°C, 0°C and -12°C. The bars represent the standard errors calculated from three biological replicates of each experiment.

All the *G*. *antarctica* AFP sequences were predicted to be extracellular proteins due to the presence of N-terminal signal peptides. This is expected due to the defensive role of AFPs in manipulating the environment surrounding the cell to prevent ice formation. Although it is probable that expression levels of the AFPs are influenced by temperature-dependent protein stability [42], the availability of different AFPs can in itself be regarded as an important clue regarding their potential roles as responses to different types of ice structures. The interactions of AFPs with ice (reviewed by Jia and Davies—[43]) are similar to other protein-ligand interactions, albeit unusual in that they involve extensive Van der Waals or hydrophobic contacts that are supplemented by hydrogen bonds. Because ice formation can result in different planes of ice that may have different surface topology and spacing between the oxygen atoms, these resulting different ice surfaces can therefore be regarded as different ligands that will interact with different AFPs. Our experiments to quantify the expression levels of the *G*. *antarctica* AFPs revealed that the expression levels of all putative AFP genes was generally higher at lower temperatures (0°C—12°C) (Fig 2).

At 0°C, we observed that the expressions of *GaAFP3* increased only after 48 hours. However in the -12°C experiments, expression of *GaAFP3* was highest at the 6 hour sampling point. Some of the genes were found to be highly expressed during the early stages of exposure (6 hours, -12°C) had decreased levels of expression over time (Fig 2). Our results also showed that *GaAFP3* was the most highly expressed of the *GaAFP* genes at -12°.

We also found a consistent relationship between the expressions of Ga*AFP*1, Ga*AFP*7 and Ga*AFP*8, which are adjacent in the *G*. *antarctica* genome (Fig 2). The expression of Ga*AFP*1 seems to be reciprocal to that of the other two members of this cluster, being highest when expression of Ga*AFP*7 and 8 is lowest, and vice versa. We also find that Ga*AFP*7 and Ga*AFP*8 are expressed sequentially, with the former being more highly expressed until 72 hours, after which the latter becomes more highly expressed, under all conditions of cold-stress.

The proximity of the genes encoding *GaAFP1*, *GaAFP7* and *GaAFP8* to each other suggests a shared regulatory mechanism that may involve a sensor system that triggers a different type of AFP to be produced. In this case, the sensor system may be shared but yet able to differentiate different types of ice structures and the reporting of the system results in the different expressions observed for GaAFP7 and GaAFP8. In bacteria, cold temperature sensing is believed to involve a membrane associated signal transduction system and these systems were reviewed by Shivaji et al [44]. Although quite likely, it is unknown if a similar system is also applicable to yeasts. Changes in membrane fluidity that may change the expression of the *DES* genes, which we discuss later, may have a correlation to the *GaAFP* expression. However, we are unable to make the correlation at this time due to the depth of our data not allowing for such relationships to be ascertained. The time point associated trigger for AFP8 may be as simple as a detectable change in the ice structures encountered where over time, the ice structures begin evolving a different structure. The consistency of this 72 hour time mark may therefore be a result of the ice developing under the constant solute and temperature conditions of the experiment and may not reflect the actual response of the gene expression over time in the natural sea ice environment of *G*. *antarctica*.

Hew et al [45] reported that the expression of AFP proteins from salmon is related to the seasonal temperature, with the highest levels of AFP expression detected in November (winter) and minimum levels in May (summer). The range of temperatures that the *G*. *antarctica* yeasts were subjected to in our experiments can be viewed as simulative of the seasonal temperature changes that may happen in the Antarctic sea water where the highest levels of expression were detected at -12°C while at the same time, the data suggests different roles for the different AFPs, which in turn reflects the possibility of different AFPs targeting different planes of the ice crystals or different types of ice formations. We further investigated this possibility by conducting thermal hysteresis assays and observing the ice recrystallization inhibition by the different GaAFPs.

### Thermal hysteresis and ice recrystallization inhibition activity of *G*. *antarctica* AFPs

Full length cDNAs were generated from transcripts of the AFPs that were extracted from 4°C. These cDNAs were cloned and heterologously expressed by *E*. *coli* for ice-recrystallization inhibition and thermal hysteresis assays. All nine *G*. *antarctica* AFPs were overproduced and their thermal hysteresis activities assayed. Thermal hysteresis is the effect created by AFPs which can be measured as the difference between the melting point and the freezing point. The highest TH values recorded was for GaAFP4 (TH = 0.079±0.008°C) while the lowest was that of GaAFP5 (TH = 0.034±0.008°C).

**Fig 3:**
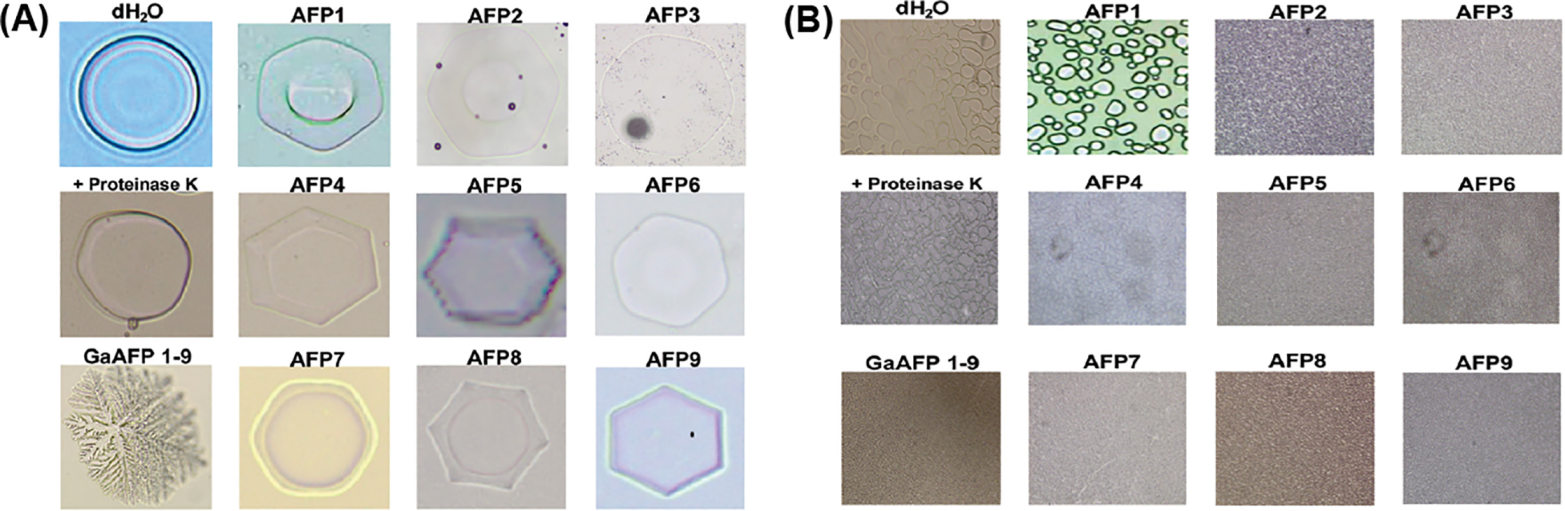
Antifreeze activity exhibited by recombinant GaAFP. (A) Ice crystals in samples containing recombinant GaAFP proteins were deformed into hexagonal shapes while the control untreated sample of ice crystals were disk shaped and the AFP eliminated samples containing proteinaseK reverted to the circular shape of the untreated sample; (B) ice recrystallization inhibition activity after incubation at -6°C for 30 min where samples containing the nine recombinant GaAFP proteins resulted in the formation of smaller ice grains compared to the coarse grains observed for the untreated samples while the samples that have the AFP effect eliminated by proteinaseK have reverted to coarse ice grains.

Ice-crystals formed on the individual recombinant proteins were modified into a hexagonal shape (Fig 3A) while the ice crystals in the control sample that were not treated with any *G*. *antarctica* AFPs remained a circular disk shape. A cocktail of all recombinant GaAFPs resulted in TH activity (0.147±0.005°C) that was slightly higher than those recorded by Hashim et al [41] using wild-type proteins (0.1°C). The qualitative recrystallization inhibition activity assays revealed that the ice grains in samples containing GaAFP1 and GaAFP5 were smaller (Fig 3B). Mixtures of all recombinant AFPs changed the ice-crystals to a star-like shape that resembled those formed with wild-type proteins. The addition of proteinase K to all recombinant AFPs eliminated the inhibition of ice-crystal formation thus proving that the observed hysteresis activity was a result of the AFPs’ activity. Based on a review of the available literature, the work we report here is the first time in which all the identified AFPs from a single organism have been experimentally confirmed.

The presence of multiple genes encoding different AFPs has been well documented (see Background section). However, our gene expression analysis over time and at different temperatures, in addition to the thermal hysteresis and ice recrystallization inhibition assays, were further able to provide possible context with regard to the requirement for multiple AFP genes. It is clear that the various AFPs are not expressed simultaneously but are instead produced as seeming responses to specific temperatures and time periods for a sustained temperature. Although we do not have direct evidence of association, this observation implies that different AFPs may interact with different ice structures that form at different temperatures; furthermore ice structures at the same temperature may also change over time thus requiring different AFPs to be deployed as a response over a longer time period at the same temperature.

### Fatty acid desaturases

**Fig 4:**
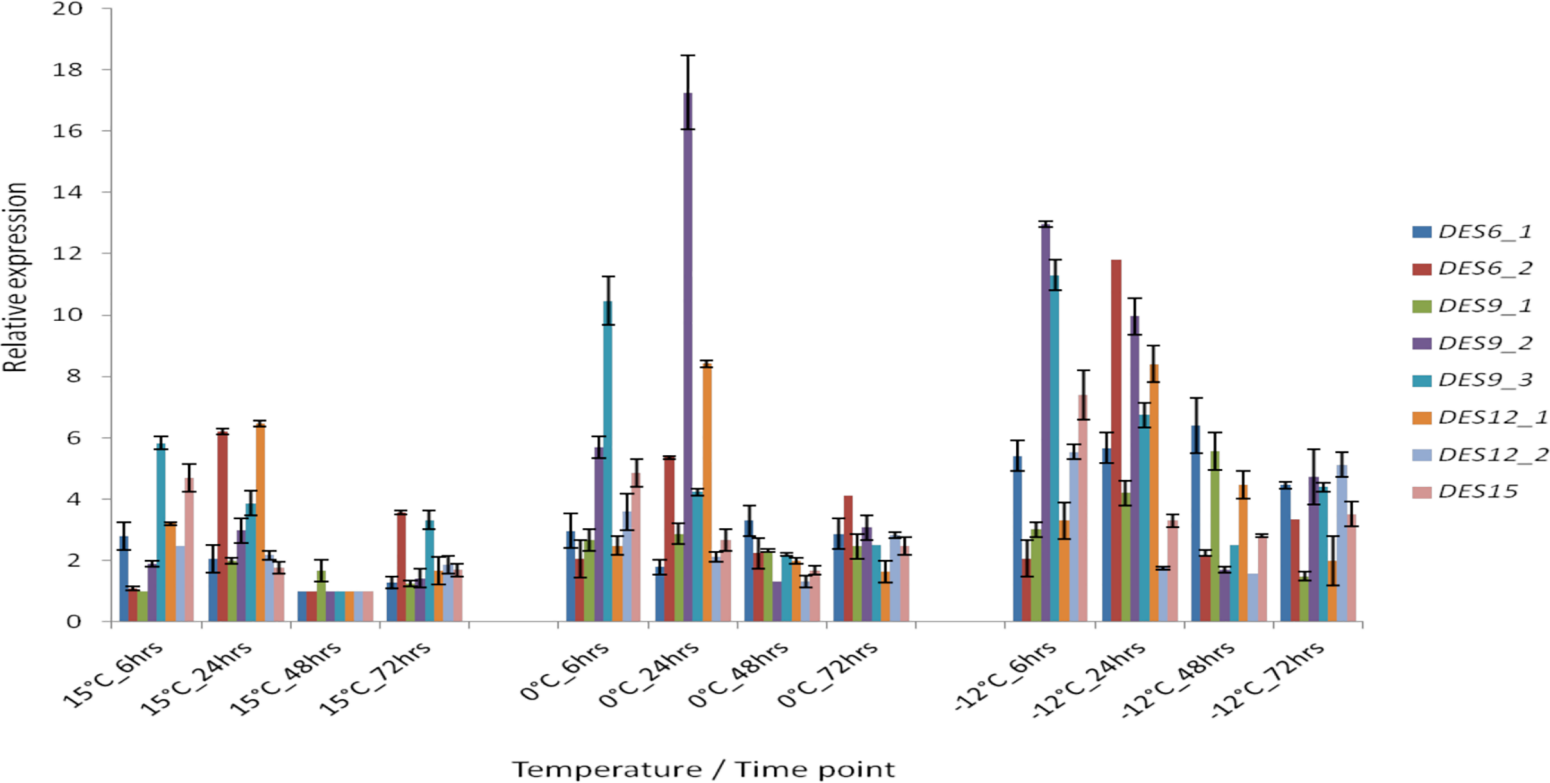
Relative expression profile of fatty acid desaturases (FADs) at different growth temperature; 15°C, 0°C and -12°C. The bars represent the standard errors calculated from three biological replicates of each experiment.

The synthesis of unsaturated fatty acids by fatty acid desaturases (FAD), enzymes that introduce double bonds into membrane lipids, has been shown to be up-regulated in response to low temperatures in cyanobacteria [46], milkfish and grass carp [47] and insects [48]. A total of eight FADs, from four different families (DES6, DES9, DES12 and DES15) were annotated for *G*. *antarctica*. We observed that the expression of *DES9 (DES9a;* GAN_06_293, *DES9b;* GAN_12_274, *DES9c;* GAN_12_275), *DES12a* and *DES12b* genes were higher at the early stages of exposure (6 hours, Fig 4) while the expression of *DES6* (*DES6a*; GAN_06_180, *DES6b*; GAN_11_019) and *DES15* (GAN_06_218) were higher at the later stages of exposure (48 hours, Fig 4). During the early stages of exposure, this organism will express more of the *DES9*, *DES12a* and *DES12b* genes that encode enzymes that catalyze the introduction of one or two double bonds in the fatty acid chains to increase the fluidity of the membrane. Los and Murata [49] proposed that living organisms will alter their membrane lipids by desaturating the fatty acid chains to maintain the fluidity of the biological membranes over a certain range of temperature. However, after 48 hours of cold exposure, *G*. *antarctica* was found to express more of the *DES6* and *DES15* gene products. The addition of another one double bond to the fatty acid chains will increase the fluidity of the membranes by changing its physical properties [50].

## Conclusions

The *G*. *antarctica* genome sequence has revealed multiple systems that are either known psychrophilic responses or, with the current knowledge available, appears to be present only in *G*. *antarctica*. Targeted gene expression experiments and assays further reveal that the presence of variable psychrophilic responses can be associated to time and temperature variations although the exact mechanisms for these relationships remain unclear. The temperature and time specific responses implies potentially different mechanisms may exist as response systems to the types of different sea ice structures that the organism may encounter in line with variations in temperature as a result of winter-summer transitions, temperature shifts in the ocean and variations in salinity. Shifts of expression profiles over time may be associated to the need to interact with different ice structures in the case for the GaAFPs; while for the FADs, the variations in expression may turn out to be a wear and tear avoidance and resource management mechanism required for sustained periods of cold stress such as the Antarctic sea ice habitat of this yeast.

## Methods

### Yeast isolation and culture

*Glaciozyma antarctica* PI12 was isolated on to nitrogen free Davis medium from the sea ice at Casey Station, Antarctica. Cultures were grown by subculturing cells on Yeast Peptone Dextrose (YPD: 0.1% yeast extract, 0.2% peptone, 0.2% dextrose) agar at 4°C for seven days. Cells for genomic DNA extraction were grown aerobically in YPD, containing 50 μg/mL ampicillin and 50 μg/mL kanamycin, at 4°C for six days. For cDNA library construction, cells were grown aerobically in YPD, in the presence of ampicillin and kanamycin, at 4°C for five days followed by exposure to different growth temperatures; 15°C, 4°C, 0°C and ˗12°C, for two days. The cultured cells were harvested by centrifugation at 4°C, snap-frozen in liquid nitrogen and stored at ˗80°C until further usage.

### Genomic DNA extraction

Cell pellets for genomic DNA extraction were briefly resuspended in residual liquid and 1.5 mL of cells were transferred into fresh 2 mL screw cap tubes added 0.5 mL of ddH_2_O was added and cells were centrifuged at 4°C, 23 900 *x*g for 2 min. The supernatant was decanted pellet was resuspended in residual liquid followed by addition of 0.3 g of glass beads, 200 μL of DNA extraction buffer [containing 2% TritonX-100, 1% SDS, 100 mM NaCl, 10mM Tris HCl (pH8.0), 1 M EDTA] and 200 μL of phenol:chloroform:isoamyl alcohol (25:24:1). The tube was vigorously vortexed for 15 min followed by addition of 200 mL TE buffer. Cells were snap-frozen in liquid nitrogen for 1 min and transferred into 65°C water bath for 3 min. Tubes were centrifuged at 23 900 *x*g for 4 min and the aqueous layers were transferred to fresh tubes. Subsequently, 1 mL of 100% ethanol was added, mixed and centrifuged at 23 900 x*g* for 5 min. The pellets were resuspended in 0.4 mL of TE, 5 μL of RNAse A (10 mg/mL) was added and incubated at 37°C for 15 min. Precipitation was performed using 10 μL of 4 M ammonium acetate and 1 mL of 100% ethanol. The DNA was pelleted by centrifugation for 5 min at 23 900 *x*g, washed with 70% ethanol, air dried and resuspended in 50 μL of ddH_2_O.

### Construction and sequencing of cDNA libraries and ESTs

Approximately 1 g of powderized snap frozen yeast cells were homogenized in 10 mL TRIZOL reagent (Invitrogen, USA). RNA was then extracted following the standard manufacturer’s instructions. RNA pellets were resuspended in DEPC-treated water. mRNA was isolated using the PolyATtract mRNA Isolation Systems (Promega, USA) according to manufacturer’s instructions. The quality of the total RNA and mRNA extracted were verified using Bioanalyzer (Agilent Technologies, USA). A total of 3 μg of mRNA was used to construct cDNA library using the CloneMiner cDNA Library Construction Kit (Invitrogen, USA) as per the manufacturer’s protocol. Individual colonies from each library were transferred to 96-well microtiter plates for sequencing and storage. The insert sizes for randomly picked clones were estimated by digestion with *Bsr*G1 restriction enzyme. Large scale plasmid extraction was performed using the Montage Plasmid Miniprep Kit (Millipore, USA) according to standard manufacturer’s protocol. The extracted plasmids were eluted with ddH2O and sequenced using the ABI BigDye Terminator v3.1 (Applied Biosystems, USA) on 3730xl DNA sequencer (Applied Biosystems, USA). All of the clones were sequenced from one end using M13 forward primer; 5’-GTAAAACGACGGCCAG-3’. The quality of the generated sequences was assessed using Phred and the vector sequences were trimmed using Crossmatch software. Sequences were clustered and analyzed using StackPACK v2.2.

### Fosmid library construction

The genomic DNA was prepared via the Hoffman and Winston method [51]. The DNA was then sheared by repeated aspiration and expulsion of the samples from the pipette tip (50–100 times). The DNA fragments were then isolated for a range of 25–50 kb via electrophoresis in a 20 cm long, 1% agarose gel at 30 V for 16 hours. The DNA products were end-repaired with the end-repair enzyme mixtures included in the CopyControl™ Fosmid Libary Production kit (EPICENTRE: Madison, Winconsin) and then ligated into fosmid vector PCC1FOS according to manufacturer's protocol. Packaging of the fosmid clones were done using MaxPlax Lambda Packaging Extracts (EPICENTRE: Madison, Winconsin). Packaged fosmids were stored at 4°C with the addition of chloroform and phage dilution buffer (10 mM Tris-HCl [pH 8.3], 100 mM NaCl, 10 mM MgCl_2_). Serial dilutions of packaged fosmid clones (phage particles) were done to calculate the clone titer. Phage particles were mixed with EPI-T1 cells in a ratio of 100 μL cells for every 10 μL of diluted phage particles. These mixtures were then incubated at 37°C for 20 min. Infected bacteria were then spread on LB plate + 12.5 μg/mL chloramphenicol and incubated at 37°C overnight to select for fosmid clones. Induction of fosmid clones were then carried out according to the manufacturer's protocol using 1000X CopyControl induction solution.

### Genome pyrosequencing and assembly

The *G*. *antarctica* genome was sequenced using a Genome Sequencer FLX sequencing instrument (454 Life Sciences, Branford, CT). Three sequencing rounds with an additional sequencing round for paired ends were carried out. Assembly was performed by using the Newbler assembler software version 1.1.02.15.

### Gene prediction and sequence analysis

The genome assembly was annotated using a combined approach. Gene prediction was carried out using GeneMark-ES, Augustus, and GlimmerHMM. GeneMark-ES predictions were trained using EST validated open reading frame (ORF) predictions (see below) and *ab initio* runs. Augustus and Glimmer predictions were trained using datasets from *Cryptococcus neoformans*. EST sequences were also mapped to the assembled genome sequence and matched to the closest ORF.

### Genome annotation

All predicted gene models were annotated for using InterProScan [52] and BLAST alignments [53] against the InterPro [54], UniProtKB/SwissProt [55, 56], KEGG (Kyoto Encyclopedia of Genes and Genomes) [57, 58] and Pfam [59, 60] databases. InterPro hits were mapped to Gene Ontology terms. ORFs that did not correspond to any database hits were annotated a hypothetical proteins. All scaffolds, gene models and annotations are available via the Malaysia Genome Institute *G*. *antarctica* genome browser (http://mfrlab.org/glacier/) and under the GenBank accession number ASRT0000000. The gene models were further analysed using a variety of programs to identify potential membrane proteins and signal peptides.

Intergenic and intronic sequences were extracted from the assembled scaffolds using the Artemis genome browser [https://www.sanger.ac.uk/resources/software/artemis/] [61]. The SNOSCAN program was utilized to search for guide box C/D snoRNAs candidates targeting putative rRNA methylation sites. *G*. *antarctica* ribosomal RNA sequences and their methylation sites were compiled and fed into the SNOSCAN program for *guide* box C/D snoRNA prediction. Hits with scores higher than 20 and in the correct orientation were selected for further analysis. Both the SnoReport and SnoSeeker programs were employed to locate potential *orphan* box C/D snoRNAs. Output generated was compared and contrasted by using VLOOKUP function in Excel to identify similar hits generated by both SnoReport and SnoSeeker program.

### Gene expression analysis by qRT-PCR

#### Growth conditions

*G*. *antarctica* cells were cultured in 50 mL Yeast Peptone Dextrose (YPD) at 4°C for 5 days at 180 rpm, then exposed at different temperatures: 15°C, 0°C and -12°C for another 6 hours, 24 hours, 48 hours and 72 hours. The cells were harvested by centrifugation at 1000 *x*g for 5 min and then snap frozen in liquid nitrogen and stored at -80°C.

#### Total RNA extraction

Total RNA was prepared using TRIZOL reagent (Invitrogen, USA) according to the standard manufacturer’s instructions. The ratio between cells and reagent is is 0.1 g cells to 1 mL reagent. Final RNA pellets were re-suspended in DEPC-treated water and purified using the RNeasy Mini Kit (Qiagen). Purified RNA quality was verified by (i) running the total RNA in 1% agarose gel electrophoresis, using the Bioanalyzer RNA Nanochip (Agilent Technologies) and (iii) using Nanodrop Spectrophotometer ND-1000 (absorbance ratios, A_260/280_). Purified RNA with RNA Integrity Number (RIN) between 9.0 to 10.0 were then used for the quantitative real-time polymerase chain reactions (RT-qPCR).

#### PCR primers

Primers for RT-qPCR were designed using the Primer Premier 5 software and synthesized by First Base Laboratories Sdn. Bhd. (Malaysia).

#### Quantitative real-time PCR

The RT-qPCR reactions were carried using a Mastercycler^®^ ep *realplex* (Eppendorf). The assays were run by using Quantifast SYBR Green RT-PCR kit (Qiagen). Each real-time PCR mixture (final volume 20 μL) contained 10 μL of QuantiFast SYBR Green RT-PCR Master Mix, 0.25 μL of a Reverse Transcription mix, 0.25 μL of a 25 μM forward/reverse primer, 1 μL of RNA template (~100 ng), and 8.25 μL of RNase-free water. Specific primers were designed based on sequence data from *G*. *antarctica* database (Table B in S1 File). Three biological samples for each temperature and corresponding sampling time point were each also replicated three times and run using a one-step RT-PCR with 50°C for 10 min, 95°C for 5 min, followed by 40 cycles of 95°C for 10 s, and 60°C for 30 s. At the completion of each run, melting curves for the amplicons were measured by raising the temperature by 0.4°C from 60°C to 95°C while monitoring fluorescence. PCR amplification was assessed from the melting curve derivative for Tm, its symmetry and the lack of non-specific peaks. PCR amplification was confirmed by 2% agarose gel electrophoresis and melting curve analysis.

### Data and statistical analysis

All data analyses were performed with GenEx (version 4.4.2.308, MultiD Analyses). The gene expression ratio was recorded as the relative expression level from samples that grew at 15°C, 0°C and -12°C. The results were normalized against a reference gene, 18S rRNA. All the statistical analyses were performed using Minitab 15. Two-way analysis of variance (ANOVA) at a significance level of P < 0.05 was used to compare the expression levels at different temperatures and exposure periods.

### Functional characterization of recombinant GaAFPs

#### cDNA amplification, cloning into expression vector and transformation

Total RNA of *G*. *antarctica* was prepared using Trizol reagent (Invitrogen/Life Technologies, Carlsbad, CA, USA). First strand cDNA of *G*. *antarctica* was synthesised using the SuperScript^®^ III First-Strand Synthesis System (Invitrogen) based on the manufacturer’s protocol. Then, the first strand was used as template for full length cDNA amplification. Specific primers were designed based on sequence data from *G*. *antarctica* database (Table B in S1 File). To express heterologous proteins in *E*. *coli*, sets of primers were designed to amplify the mature coding sequence with specific restriction enzyme sequences added for ligation into expression vectors. A total of 50 μL of PCR reaction mixture contained: 2 μL of first strand cDNA, 1 X PCR Buffer, 0.2 mM dNTP, 20 pmol/μL forward and reverse primers, 1.5 mM MgCl_2_, 0.5 U/μL *Taq* polymerase (Invitrogen). Initial denaturation was at 94°C for 5 min followed by 29 cycles of denaturation at 94°C for 45 s, annealing at 60°C for 30 s and elongation at 72°C for 1 min with final extension at 72°C for 15 min. The PCR products were analysed by agarose-gel electrophoresis (1% w/v) and cloned into the pGEMT-Easy Vector System (Promega, Fitchburg, Madison, WI, USA). Subsequently, the cDNAs were subcloned into expression vector pET28b (Merck, Darmstadt, Germany) and transformed in *E*. *coli* DH5α as well as the expression host BL21 (DE3). Plasmids were extracted from selected positive colonies and their DNA sequenced.

#### Heterologous expression in *E*. *coli*

A single colony of *E*. *coli* BL21 (DE3) carrying positive transformants of pET28_Afp was grown in 5 mL Luria Bertani (LB) medium containing kanamycin (37°C, overnight). The culture was pelleted and re-suspended in fresh LB broth. Approximately 1% of starting culture was transferred to 50 mL LB broth supplemented with kanamycin (30 μg/mL). Cells were grown at 37°C at 200 rpm with shaking to an OD_600_ of ~ 0.5–0.6. Subsequently, cultures were induced by addition of 1 mM isopropyl-β-D-thiogalactosidase (IPTG). Cells were grown for an additional 6 h at 37°C, 200 rpm and harvested by centrifugation.

#### Preparation of inclusion bodies, refolding and purification of recombinant GaAFPs

Harvested cells were re-suspended in lysis buffer (10 mM Tris pH 7.5, 150 mM NaCl, 5 mM EDTA) and disrupted by three cycles (5s each) of sonication. Cell debris then was treated with 20 μg/mL DNaseI, 3 mM MgCl_2_, 10 mM DTT and 1% (v/v) Triton X-100 before centrifugation at 10 600 x*g* for 15 min at 4°C. The supernatant was discarded and the pellet used for the preparation of inclusion bodies (IB).

Pellets were washed three times using Wash Buffer I (50 mM Tris pH 7.5, 0.5% (v/v) Triton X-100, 100 mM NaCl, 1 mM EDTA, 1 mM DTT, 0.2 mM PMSF). The samples were homogenised and centrifuged at 10 600 *x*g for 15 min at 4°C. To remove Triton X-100, the pellet was washed once with Wash Buffer II (50 mM Tris pH7.5, 100 mM NaCl, 1 mM EDTA, 1 mM DTT, 0.2 mM PMSF) and centrifuged at 10 600 *x*g for 15 min at 4°C. IBs were solubilised in 10 mL per litre culture using Solubilization Buffer (8 M Urea, 20 mM Tris pH 7.5, 150 mM NaCl, 0.5 mM EDTA, 1 mM EDTA) and centrifuged at 16 000 *x*g for 30 min at 4°C. The supernatant containing solubilised IB was harvested and stored at– 80°C until further use.

Refolding of recombinant GaAFPs was carried out using the drop-wise dilution method. The protein solutions were diluted into refolding buffer (10 mM Tris pH 7.5, 150 mM NaCl, 0.5 mM oxidised gluthatione, 5 mM reduced gluthatione, 2 mM EDTA, 0.2 mM PMSF to a final concentration of 0.05 mg/mL. Different additives were used for each GaAFP during refolding which is 0.4 M arginine for GaAfp3, GaAfp4 and GaAfp5, 10% (v/v) glycerol GaAfp7 and GaAfp9 and 5 mM β-cyclodextrin for GaAfp1, GaAfp2 and GaAfp6. All samples were stirred at 4°C for 24 h.

Refolded proteins were purified using Ni NTA agarose pre-equilibrated with 10 mM Tris pH7.5, 150 mM NaCl. The recombinant proteins were eluted from the gels using 10 mM Tris pH7.5, 150 mM NaCl, 500 mM imidazole. Eluted fractions were pooled and concentrated using 10 kDa Amicon ultracentrifuge (Merck-Millipore, Darmstadt, Germany). The purified proteins were further purified by gel filtration chromatography via Superdex S75 10/300 column (GE Healthcare, USA) using ÄKTAPurifier System (GE Healthcare, USA)

#### GaAFP activity assays

AFP activity was measured using both the ice re-crystallization inhibition (RI) assay and the thermal hysteresis assay (TH) [62]. Protein concentrations were standardised to 5 mg/mL. Samples were analysed using a temperature-controlled freezing stage (Model THM 600, Linkham Scientific Instruments, Chilworth, Guildford, Surrey GU4 8RU, UK) with a temperature controller programming unit (Model TMS 94, Linkham Scientific Instruments). The freezing stage was placed onto the stage of a conventional microscope (Olympus Corporation model BX51, Tokyo 163–0914, Japan). To determine thermal hysteresis activity, a 1 μL sample (5 mg/mL) was applied to a circular glass slide. The stage remained at 20°C, and the sample was cooled to −40°C at a rate of 100°C/min and later heated to −5°C at the same rate. The sample was warmed slowly at rate 1°C/min until a single ice crystal of >10 μm was observed. The temperature was then lowered slowly to allow observation of ice-crystal growth. In the absence of AFP, ice crystals were round and flat; however, ice crystals were irregularly shaped in the presence of AFP.

The assay for ice re-crystallisation was performed using a mixture of AFP solution and 30% (w/v) sucrose (1:1 with a final protein concentration of 5 mg/mL) that was ‘sandwiched’ between two circular glass cover-slips. The sample was then cooled to −80°C and maintained at −6°C. Observations were conducted after 30 min for ice re-crystallisation in the presence of AFP compared to a control sample (distilled water only).

To look at the effect of the mixtures on TH value GaAFP, a total of 5 mg/mL of each recombinant GaAFP were mixed in a tube. Solutions were then diluted to final concentration of 5 mg/mL and 1 μL were taken from it for activity assay. Effect of proteinase K was observed to look whether the activity came from the recombinant. Proteinase K (Sigma, USA) was added to a final concentration of 1 mg/mL and then incubated at 20°C for 30 min. Samples were taken after incubation to look for AFP activity.

## Supporting information

S1 File (Table A) Parameters for *G*. *antarctica* growth conditions (Table B) Primers used in the RT-qPCR analysis.
(PDF)
