# Supplementary material for: The Glaciozyma antarctica genome reveals an array of systems that provide sustained responses towards temperature variations in a persistently cold habitat
Source: PLoS One. 2018 Jan 31;13(1):e0189947. doi: 10.1371/journal.pone.0189947 (PMC5791967; doi:10.1371/journal.pone.0189947)
Supplement: S1 File — (Table A) Parameters for G. antarctica growth conditions (Table B) Primers used in the RT-qPCR analysis. (PDF) [file pone.0189947.s001.pdf]

Table A: Parameters for *G. antarctica* growing condition

	Temperature (°C)	Days	Exposure temperature (°C)	Days	Medium
Different exposure temperatures	4	5	15<br>0<br>-12	2	YPD (Yeast Peptone Dextrose)
Different growth medium	4	7	-	-	YNB (Yeast Nitrogen Base)<br>YPD
Different growth phases	4	2<br>5<br>8<br>12	-	-	YPD

Table B: Primers used in the RT-qPCR analysis

Genes	Primer sequences (5'-3')
Delta-6a fatty acid desaturase (*DES6a*)	(F) TGAGTCTCGGTTGGTGGAT
	(R) GAGGAACTGAGTGGGCTTG
Delta-6b fatty acid desaturase (*DES6b*)	(F) TCTCGTCTACCGAATCCC
	(R) CGTTGCTCGCATCCC
Delta-9a fatty acid desaturase (*DES9a*)	(F) ACCTCCTCGGATTCGCTAC
	(R) GGACGCTTCTTGGAACTCA
Delta-9b fatty acid desaturase (*DES9b*)	(F) GCGAAGGCGGGAATGA
	(R) CGAGAAGCAGGACCAAAGG
Delta-9c fatty acid desaturase (*DES9c*)	(F) CGGACGATGTGGCTCTG
	(R) GGGAACGACGCTTGGA
Delta-12a fatty acid desaturase(*DES12a*)	(F) CCCTATCGGTCCCTACATC
	(R) GCGGCAAGTCCTCTGGTT
Delta-12b fatty acid desaturase (*DES12b*)	(F) GCGGGTGGAACTTTGT
	(R) GTGCTTGGAGTGGGAGA
Delta-15 fatty acid desaturase (*DES15*)	(F) TACTACGGTCCGCTCAACA
	(R) CTACTCCAAAGCCCAACG
Anti-freeze protein 1 (*AFP1*)	(F) CTCCCTGCTCGTCAAAG
	(R) GCCAGCGAAGTCCCTCT

Anti-freeze protein 2 (*AFP2*)	(F) CCTCCGCAAAGGTCATTC
	(R) AGCATAGGCGTTTCCAGT
Anti-freeze protein 3 (*AFP3*)	(F) ACGCTACCAACATCG
	(R) GAAGGGAGCAACAAC
Anti-freeze protein 4 (*AFP4*)	(F) GTGTCGGAAAGGGAGC
	(R) GGGACGAAGGGAGAGTT
Anti-freeze protein 5 (*AFP5*)	(F) CTCGTCTCTCTCCTCGTCG
	(R) GCAGGGGAAACTCCAATG
Anti-freeze protein 6(*AFP6*)	(F) TCTACGAGCCCGACCCA
	(R) CCGTGACCGTGATGAGT
Anti-freeze protein 7 (*AFP7*)	(F) CTCACCGATTATGCC
	(R) CAAAGTCCTGCCACC
Anti-freeze protein 8 (*AFP8*)	(F) CGACTACTGTTCGGGAGAC
	(R) GAAGAGCGTGACGACTGAC
Anti-freeze protein 9 (*AFP9*)	(F) TTCACCGTTCAAGCCCC
	(R) GGAGACAAATCCTTCCACAG
